# Visual processing in reading disorders and attention-deficit/hyperactivity disorder and its contribution to basic reading ability

**DOI:** 10.3389/fpsyg.2015.01635

**Published:** 2015-10-27

**Authors:** Michelle Y. Kibby, Sarah M. Dyer, Sarah A. Vadnais, Audreyana C. Jagger, Gabriel A. Casher, Maria Stacy

**Affiliations:** Department of Psychology and Center for Integrated Research in Cognitive and Neural Sciences, Southern Illinois UniversityCarbondale, IL, USA

**Keywords:** dyslexia, reading disability, reading disorder, ADHD, children, basic reading, visual processing, visual short-term memory

## Abstract

Whether visual processing deficits are common in reading disorders (RD), and related to reading ability in general, has been debated for decades. The type of visual processing affected also is debated, although visual discrimination and short-term memory (STM) may be more commonly related to reading ability. Reading disorders are frequently comorbid with ADHD, and children with ADHD often have subclinical reading problems. Hence, children with ADHD were used as a comparison group in this study. ADHD and RD may be dissociated in terms of visual processing. Whereas RD may be associated with deficits in visual discrimination and STM for order, ADHD is associated with deficits in visual-spatial processing. Thus, we hypothesized that children with RD would perform worse than controls and children with ADHD only on a measure of visual discrimination and a measure of visual STM that requires memory for order. We expected all groups would perform comparably on the measure of visual STM that does not require sequential processing. We found children with RD or ADHD were commensurate to controls on measures of visual discrimination and visual STM that do not require sequential processing. In contrast, both RD groups (RD, RD/ADHD) performed worse than controls on the measure of visual STM that requires memory for order, and children with comorbid RD/ADHD performed worse than those with ADHD. In addition, of the three visual measures, only sequential visual STM predicted reading ability. Hence, our findings suggest there is a deficit in visual sequential STM that is specific to RD and is related to basic reading ability. The source of this deficit is worthy of further research, but it may include both reduced memory for order and poorer verbal mediation.

## Introduction

How much visual processing contributes to reading disorders, and to reading performance in general, has been a topic of debate for decades. Whereas some early theorists suggested reduced sensory-perceptual processing plays a pivotal role in reading disability (RD)/developmental dyslexia (DD) (Kephart, 1960; Wepman, 1964; Boder, 1973; LaBerge and Samuels, 1974), others have argued that DD/poor reading ability is due to linguistic-based deficits and not sensory-perceptual ones (Bradley and Bryant, 1978; Hammill and McNutt, 1981; Stanovich, 1985; Ramus et al., 2003; Vellutino et al., 2004; Ziegler et al., 2010). Nonetheless, some researchers continue to find visual processing deficits in DD/poor readers today (Facoetti et al., 2008; Vidyasagar and Pammer, 2010; Germano et al., 2014). To address this controversy, our study examined three aspects of visual processing [perception (discrimination of complex figures), short-term memory (STM) for complex figures, STM for sequences of basic shapes] to determine whether they are affected in children with RD. As ADHD is frequently comorbid with RD, children with this disorder were included as a comparison group, along with children who have both disorders and typically developing controls. Moreover, in order to determine whether visual processing plays a role in the variability of reading performance, this study also examined whether visual processing predicted basic reading ability in the total sample after controlling for phonological or orthographic processing.

Multiple researchers have found various deficits in visual processing in DD recently. These deficits include reduced multi-character processing/visual attention for briefly presented stimuli (Hawelka et al., 2006; Bosse et al., 2007; Dubois et al., 2007; Lassus-Sangosse et al., 2008), visual discrimination of quickly presented items (Ortiz et al., 2014), visual closure and form constancy (Germano et al., 2014), and visual search (Jones et al., 2008). Nevertheless, spatial processing of visually-presented material may be intact (von Károlyi, 2001; Rusiak et al., 2007; Brunswick et al., 2010), and the visual-spatial sketchpad/visual STM may be intact when central executive and rapid visual attention demands are low (Kibby et al., 2004; Smith-Spark and Fisk, 2007; Kibby and Cohen, 2008; Swanson et al., 2009; Bacon and Handley, 2014; see Kibby, 2012 for a review). However, there may be STM deficits in DD when items need to be recalled in order/sequentially, in contrast to when the focus is on STM for a single item (Perez et al., 2012). Having two or more deficits that may lead to dyslexia (e.g., visual, phonological, and/or orthographic processing deficits) is consistent with the notion that dyslexia is a heterogeneous disorder in its etiology (Pennington, 2006; Kibby, 2009; Menghini et al., 2010).

A meta-analysis was performed by Kavale and Forness (2000) to determine how well visual and auditory perception predicted various aspects of reading achievement. They clustered visual measures into seven types: visual discrimination, visual closure, visual spatial relationships, figure ground discrimination, visual association, visual-motor integration, and visual memory (lumping various forms of this together including sequential and non-sequential STM). They found visual memory and visual discrimination to be the best predictors of general reading ability, and word recognition in particular, when analyzing the visual measures. The authors also directly compared auditory and visual perception measures as predictors of reading ability, along with IQ, using stepwise regression. IQ was the first variable entered, and it explained about half of the variance in general reading ability and in word recognition. For general reading ability, visual discrimination, visual closure, and visual memory were entered by the regression next, explaining an additional 7% of the variance. For word recognition, the second variable entered was an auditory skill (auditory memory), followed by visual discrimination and visual memory. For both equations, the remaining variables explained only 2–3% more variance. Based upon their findings Kavale and Forness concluded that including perceptual skills in an analysis helps to increase the accuracy of predicting reading achievement, but how much it does so depends upon the combination of variables included in the analysis (visual, auditory, or both) and whether IQ is used as a covariate. When including IQ as a covariate, they believed perceptual processes added limited predictive power and should no longer be considered as primary factors in predicting reading ability. Nonetheless, it is unknown how much visual (and auditory) perception the IQ measures required. Thus, IQ may not have been a good covariate. Taking the various findings presented together, they suggest to the current authors that visual memory and visual discrimination, and perhaps visual closure, warrant further investigation in terms of how well they predict reading ability and whether they are affected in RD.

Along with determining that certain visual processing abilities may be predictive of reading achievement, researchers have demonstrated a possible route for this. Early research suggested that visual perception and visual STM play an important role in reading, especially when taking a “whole word” approach (Boder, 1973). Later on, as the field progressed, visual processing was linked to orthographic processing and the orthographic route to reading in particular (Corcos and Willows, 1993; Stein and Talcott, 1999; Au and Lovegrove, 2006). Mesman and Kibby (2011) analyzed potential predictors of orthographic processing (exposure to print, rapid automatized naming, and visual processing) based on various theories to determine their relative contributions to orthographic processing. Via hierarchical regressions, they found that exposure to print, rapid naming, and visual processing [a composite of discrimination, STM for complex figures presented singularly, and STM for sequences of basic shapes using the Test of Visual Perceptual Skills-Revised (TVPS-R)] each predicted orthographic functioning regardless of their position in the equation, even when controlling for phonological awareness and vocabulary knowledge, which also were significant. Hence, a link was demonstrated between visual processing and orthographic processing, even when controlling for various aspects of linguistic processing. What is unknown is whether there would have been dissociable contributions between the various aspects of visual processing assessed.

ADHD is frequently comorbid with dyslexia/RD, and individuals with ADHD often have subclinical reading problems even when they do not have comorbid DD. However, the contributors to these reading weaknesses may be more variable than they are for DD. For example, Gregg et al. (2008) noted that although orthographic skills and phonological skills formed a two-factor model for the dyslexia and control groups, they did not form a good one- or two-factor model for individuals with ADHD. This was true regardless of whether they had ADHD alone or comorbid dyslexia. The authors suggested that other cognitive deficits common to ADHD, such as attention, working memory and executive functioning deficits, may be contributing to their reading weaknesses and may explain why the two factor model did not hold for the groups with ADHD.

When examining visual processing in ADHD, visual perception may be intact when spatial demands are low, but it may be poor when spatial demands are high (Aman et al., 1998; Johnson et al., 2010). This carries over to visual STM, as visual STM may be intact when spatial demands are low but impaired when spatial demands are high (McInnes et al., 2003; Karatekin, 2004; Martinussen et al., 2005; Kibby and Cohen, 2008; Kibby, 2012). Visual attention/multi-character processing may be intact in ADHD (Laasonen et al., 2012). With regard to their performance on the visual processing measure used in this study, only two studies were found that utilized the TVPS-R to study groups with ADHD (Papavasiliou et al., 2007; Crawford and Dewey, 2008). However, neither study compared participants with ADHD only to controls at baseline on the individual subtests, so it is unknown how this group would perform.

Based on the literature reviewed, visual processing, particularly visual discrimination and visual STM for sequential order, may be affected in individuals with reading disorders. In contrast, visual STM may be intact when sequencing demands are low. Nonetheless, all of these statements remain sources of debate in the literature, which is why they are investigated here. When examining ADHD the presentation is even more variable, as they may have other factors affecting their reading performance compared to controls and individuals with RD. Nonetheless, visual non-spatial processing may be intact in this group and visual-spatial processing may be impaired, suggesting dissociation from RD. Our measures of visual processing included a test of visual perception (discrimination), a test of visual STM that does not require memory for order (visual memory) and a test of sequential visual STM. As visual-spatial processing/coding requirements were limited in these tasks, individuals with ADHD were not expected to be affected and were used as a comparison group. We hypothesized that children with reading disorders (RD alone and those comorbid with ADHD) would perform worse than controls and children with ADHD on the measures of discrimination and sequential STM. We thought the Visual Memory task, which presents one geometric figure per trial, would be intact for both the RD and ADHD groups as it did not require a great deal of spatial processing nor memory for order. We also were interested in whether visual processing predicts basic reading ability after controlling for phonological processing (awareness) or orthographic processing. It was hypothesized that discrimination and sequential STM would predict basic reading ability when phonological processing was controlled but not when orthographic processing was controlled, as visual processing may be related to orthographic ability in particular.

## Materials and methods

### Participants

Participants included 264 children, ages 8–12 years. As diagnosed by a child neuropsychologist, 51 had RD, 88 had ADHD, 51 had both disorders, and 74 were typically developing children. Children with RD were identified using both a poor reader definition (below average ability on basic reading measures; < 85) and a SLD discrepancy definition following the guidelines of Pennington (2008), as there is still much debate as to which definition is best, varying by journal, field, and author. A significant discrepancy between IQ and basic reading ability was determined based on the Illinois regression formula which takes into account the distance the IQ score is from 100, such that criteria become less stringent the farther an IQ score is below 100, and more stringent the farther the IQ score is above 100. When the achievement test value required by the regression formula was lower than the value required by the poor reader definition; the poor reader criterion of < 85 was used. ADHD was diagnosed according to DSM-IV criteria, as that was the version in use at the time of data collection. Although the majority of those diagnosed with ADHD had the requisite number of symptoms according to the DSM-IV (e.g., six or more symptoms of Inattention), an exception was allowed, and a diagnosis (ADHD NOS) also was made for children with fewer symptoms if there was sufficient symptom severity to cause impairment across settings and to score a standard deviation above the mean or more on the Attention Problems and/or Hyperactivity/Impulsivity scales from the Behavior Assessment System for Children (BASC or BASC-2 depending upon the time of testing; Reynolds and Kamphaus, 1992, 2004). This exception was allowed, as children with 4–5 severe symptoms may be more impaired/have worse severity of ADHD than those with six mild symptoms given the continuous nature of attention, activity level, and impulse control. In our sample, those with ADHD NOS did not differ in symptom severity from those with six or more symptoms based on teacher report [Inattention: *t*_(128)_ = −0.21, *p* = 0.83; Hyperactivity/Impulsivity: *t*_(128)_ = −0.08, *p* = 0.94]. This sample included children with ADHD Predominantly Inattentive and Combined subtypes. ADHD Hyperactive/Impulsive type was not included as its validity after the preschool/early childhood years is debated (Marakovitz and Campbell, 1998; Barkley, 2003). Controls did not have either disorder. Exclusion criteria for all groups included other significant psychiatric or medical diagnoses (e.g., major depression, generalized anxiety disorder; any medical disorder that affects cognition), suspected or confirmed abuse, or an IQ below 80.

Our participants were from a community sample, and many were recruited as part of grant funded projects (see Section Acknowledgments). Parents brought their children to the first author's laboratory for the study. Children attended public or private schools or were homeschooled. Most children with RD had a history of intervention including remedial services, special education services, and/or tutoring. Some children with ADHD were diagnosed prior to our testing and were treated with stimulant medication, but none were on medication during testing.

### Measures

#### Intelligence

In order to control for intellectual ability, the Wechsler Intelligence Scale for Children (WISC-III or WISC-IV depending upon the time of testing; Wechsler, 1991, 2003) was administered to all participants. The third edition of this measure was used in the early stages of this study, and the fourth edition was used with later participants in order to be compliant with U.S. ethical guidelines for clinical reports and evaluations. The Verbal Comprehension Index (VCI), which includes measures of vocabulary knowledge, general knowledge typically acquired in academic settings, and abstract verbal reasoning, was used to represent verbal intellectual ability. Both the WISC-III and WISC-IV have established reliability and validity (Wechsler, 2003).

#### Attention and hyperactivity/impulsivity

In order to assist with the diagnosis of ADHD, inattention, and hyperactivity/impulsivity were measured by parent- and teacher-report using the Behavior Assessment System for Children—First or Second Edition (BASC and BASC-2; Reynolds and Kamphaus, 1992, 2004). We used the age-appropriate form (child form for ages 8–11 and adolescent form for age 12) with gender-specific norms. The Attention Problems scale from the BASC/BASC-2 measures common symptoms of inattention such as “pays attention when spoken to” and “has a short attention span.” The Hyperactivity/Impulsivity scale measures common symptoms of impulsivity and hyperactivity such as “cannot wait to take turn” and “is unable to slow down.” The parent- and teacher-report forms of the BASC/BASC-2 have good reliability and validity according to the manual.

#### Basic reading ability

The Woodcock–Johnson Tests of Achievement—Third Edition (WJ-III Form A; Woodcock et al., 2001) was used to measure basic reading ability. The subtests that comprise the basic reading cluster are Letter–Word Identification, which requires the participant to read words of increasing difficulty at our age range (8–12), and Word Attack, which requires the participant to decode pronounceable non-words. The Woodcock–Johnson Tests of Achievement has well-established reliability and validity according to the test's technical manual.

#### Phonological processing

Phonological awareness was measured using the Elision subtest from the Children's Test of Phonological Processing (CTOPP; Wagner et al., 1999). Elision requires both analysis and synthesis of phonemes, as the participant must remove the requested phoneme from a word and blend the remaining phonemes to form a new word. It is an orally administered subtest. Internal consistency for Elision ranges from 0.86 to 0.91 for 8- to 12-year-old children (Wagner et al., 1999). The CTOPP has good validity as well (Mitchell, 2001; Haight, 2006).

#### Orthographic processing

Orthographic processing was measured with a composite of two experimental measures. The first measure, adapted from Stanovich and West (1989), is a measure of homophone knowledge. The child is asked a series of questions and must select the appropriate responses from two homophone choices. The questions and the answer choices are presented in written form. The second measure, adapted from Olson et al. (1985), also measures orthographic knowledge, but in this case the child must select the real word from a pair of items, one of which is the target word and the other of which is a pseudohomophone. The pseudohomophone is not a real word but is spelled in a way that is phonologically identical to the target word. Participants are given 3 minutes to answer as many items as possible by circling the correctly spelled word in each pair. These two measures are considered fairly pure and reliable measures of orthographic functioning (Mesman and Kibby, 2011).

#### Visual processing

The three measures of perceptual processing are from the Test of Visual Perceptual Skills-Revised (TVPS-R; Gardner, 1996). Visual Discrimination is a measure of visual perception requiring discrimination between similar complex geometric figures. The participant must choose from five choices the figure that exactly matches the target figure above. Visual Memory measures immediate memory for complex geometric figures that are not easily labeled. The child has 5 s to view a figure, and then he/she must select the figure from five choices on the next page. Visual Sequential-Memory measures immediate memory for sequences of basic shapes that are readily labeled (e.g., circle, triangle, square). The shapes are presented horizontally in one line at the center of the page. The participant is given the opportunity to view the sequence for a set time period and then must select the correct sequence from four choices on the next page. As the number of items increases, the participant is given a gradually longer time period in which to view and encode the sequence. The time to view the sequence begins at 5 s for 2–3 forms and increases gradually to 14 s for 8–9 forms. These subtests scaled scores were converted to have a mean of 100 and a SD of 15 to facilitate comparison with other measures used by this laboratory. The individual subtests from this measure have demonstrated reasonable reliability and validity (Brown and Rodger, 2009).

## Procedures

All participants were administered a full day of neuropsychological tests, including the measures above, as part of grant-funded projects. Other than the WISC, complete test batteries were not administered for time reasons. Instead, subtests believed to best represent the constructs of interest were chosen. Diagnostic and other background information was gathered from participants' parents through an interview and questionnaires about their children. The Southern Illinois University Institutional Review Board's Human Subjects Committee approved the projects from which this study was derived. Before testing commenced, informed assent was obtained from all children, and informed consent was obtained from their parent/legal guardian.

## Results

### Preliminary results

To ensure groups were comparable on variables that may affect our findings, the four groups were compared on age, SES (maternal education), handedness laterality (Edinburgh), and Full-Scale IQ (FSIQ). There were no differences in ethnicity and gender when using chi-square (*p*s > 0.10). When using One-way ANOVA groups were comparable in age, maternal education, and handedness (*p*s > 0.10) but not FSIQ (*p* < 0.001). Because FSIQ includes measures that rely on visual processing, it was decided to use the VCI as the IQ covariate. Groups differed in VCI when using One-way ANOVA, *p* < 0.001. See Table 1 for descriptive data.

**Table 1:** **Participant demographic data**.

**Characteristic**	**Controls**	**RD**	**Comorbid**	**ADHD**	***df***	***X*^2^**	***p*-values**
Gender (% male)	43.24	56.86	58.82	56.82	3	4.64	0.20
Race (% Caucasian)	90.54	92.16	76.47	89.77	12	12.39	0.42
	***M (SD)***	***M (SD)***	***M (SD)***	***M (SD)***	***df***	***F***	***p*****-values**
Age (years)	9.77 (1.43)	9.53 (1.24)	9.26 (1.48)	9.55 (1.32)	(3, 260)	1.43	0.23
[95% confidence interval]	[9.44–10.10]	[9.18–9.88]	[8.84–9.67]	[9.27–9.83]			
SES (mom education)	5.69 (1.06)	5.41 (1.19)	5.32 (1.13)	5.48 (1.44)	(3, 250)	0.99	0.4
	[5.44–5.94]	[5.07–5.75]	[4.99–5.65]	[5.18–5.79]			
Edinburgh (% right handedness)	87.64 (20.59)	86.67 (18.71)	83.60 (24.14)	80.94 (26.02)	(3, 249)	1.3	0.28
	[82.80–92.48]	[81.05–92.29]	[76.74–90.46]	[75.35–86.51]			
WISC-III/IV FSIQ	105.15 (11.27)	90.59 (12.44)	87.29 (12.88)	95.59 (11.87)	(3, 260)	25.75	< 0.001
	[102.54–107.76]	[86.81–94.37]	[83.67–90.92]	[93.08–98.11]			
WISC-III/IV VCI	107.38 (13.82)	93.04 (14.68)	93.71 (14.89)	100.44 (14.17)	(3, 260)	13.88	< 0.001
	[104.18–110.58]	[88.91–97.17[	[89.55–97.87]	[93.02–97.38]			
WJ-III Reading							
Letter–word ID^a^	105.74 (10.49)	78.63 (8.55)	82.00 (8.80)	102.78 (9.87)	(3, 258)	129.42	< 0.001
	[103.31–108.17]	[76.22–81.03]	[78.50–84.50]	100.68–104.89]			
Word attack^a^	104.32 (9.56)	86.53 (6.14)	87.35 (10.26)	102.10 (9.32)	(3, 259)	67.2	< 0.001
	[102.11–106.54]	[84.80–88.26]	[84.47–90.24]	[100.12–104.09]			
Basic reading cluster^a^	105.45 (9.94)	81.57 (6.85)	83.76 (8.91)	102.67 (9.68)	(3, 258)	114.22	< 0.001
	[103.14–107.75]	[79.64–83.50]	81.23–86.29]	[100.60–104.73]			
BASC/BASC II parent report							
Hyperactivity^b^	45.29 (8.52)	46.86 (7.99)	59.72 (11.50)	60.40 (13.20)	(3, 257)	37.84	< 0.001
	[43.30–47.28]	[44.59–49.13]	[56.45–62.99]	[57.60–63.19]			
Attention problems^c^	48.40 (9.18)	53.08 (8.23)	66.66 (6.26)	67.09 (6.07)	(3, 257)	110.1	< 0.001
	[46.42–48.22]	[46.11–50.95]	[53.76–59.83]	[55.36–61.08]			
BASC/BASC II teacher report							
	46.32 (7.85)	48.53 (8.24)	56.79 (10.46)	58.22 (13.25)	(3, 244)	20.87	< 0.001
Hyperactivity^b^	[44.42–48.22]	[46.11–50.95]	[53.76–59.83]	[55.36–61.08]			
	46.57 (8.67)	53.72 (8.62)	61.71 (9.26)	61.52 (9.52)	(3, 244)	42.23	< 0.001
Attention problems^c^	[44.42–48.22]	[51.19–56.25]	[59.02–64.40]	[59.59–57.37]			

a RD and Comorbid < controls and ADHD at p < 0.001, RD commensurate with comorbid, and ADHD commensurate with controls.

b ADHD and Comorbid > controls and RD at p < 0.001, RD commensurate with controls, and ADHD commensurate with Comorbid.

c ADHD and Comorbid > controls and RD at p < 0.001, RD > controls at p < 0.05, ADHD commensurate with Comorbid.

To verify groups were comparable and disparate in functioning where expected based on diagnosis, groups were compared on basic reading measures, inattention, and hyperactivity using One-way ANOVA. Groups differed where expected see Table 1.

### Main results

**Figure 1:**
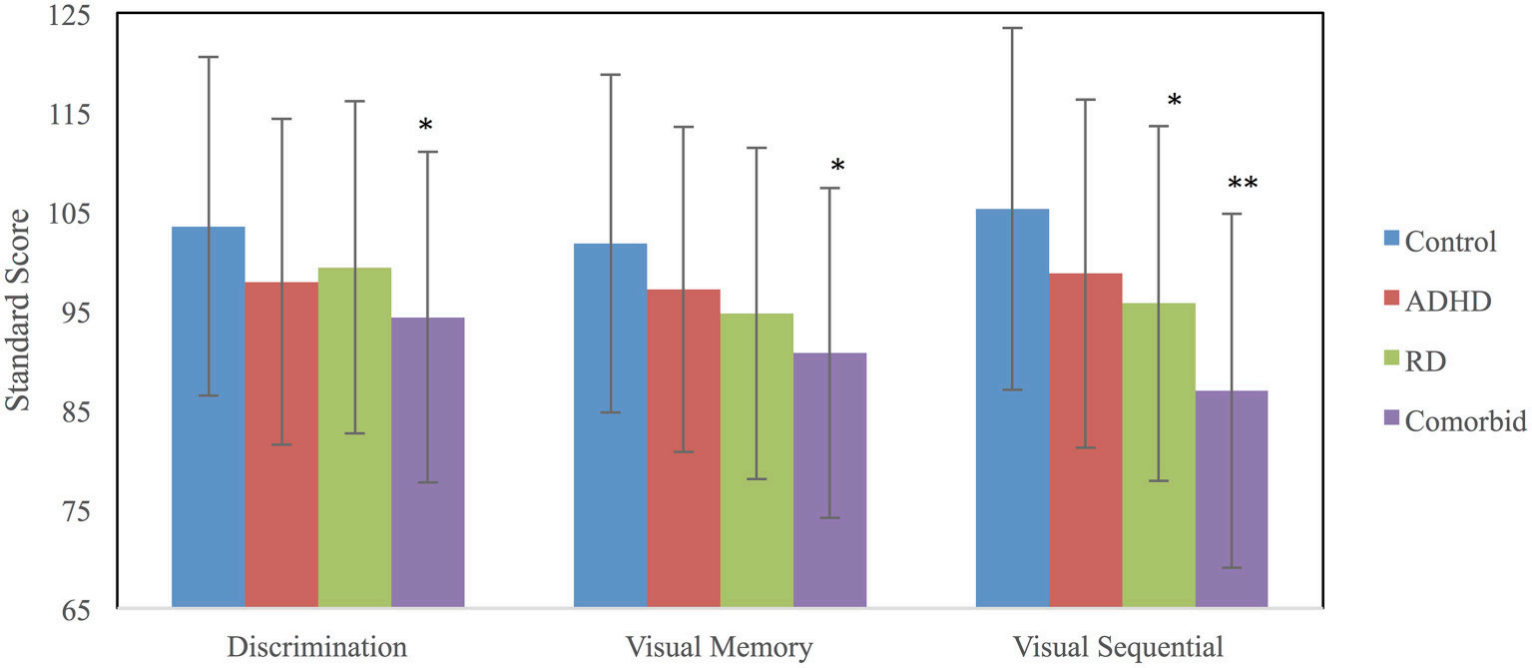
**TVPS-R marginal means for each group adjusted for the covariate, Verbal Comprehension Index (VCI)**. Error bars were derived using SD. ^*^Significantly different from the control group. ^**^Significantly different from both the ADHD and control groups.

A MANCOVA was used to compare the four groups (RD, ADHD, RD/ADHD, and controls) on the three TVPS-R measures, using VCI as the covariate. Groups differed on the visual processing measures [Wilks' Λ = 0.88, *F*_(9, 626)_ = 3.84, *p* < 0.001, $\eta_{p}^{2}=0.04$] and on VCI [*F*_(3, 260)_ = 13.88, *p* < 0.001, $\eta_{p}^{2}=0.06$] at the omnibus level. Univariate data are presented in Table 2. *Post-hoc* comparisons using SIDAK revealed that on both Visual Discrimination (*p* = 0.02) and Visual Memory (*p* = 0.003), the comorbid group performed worse than controls, whereas no other groups differed significantly from one another (*p*s > 0.10). On Visual Sequential Memory, both the comorbid (*p* < 0.001) and RD (*p* = 0.03) groups performed worse than controls, whereas the ADHD group did not differ from controls (*p* > 0.10). Furthermore, although the RD and comorbid groups were not significantly different (*p* = 0.07), the comorbid group scored significantly lower than the ADHD group (*p* = 0.001). See Table 2 for descriptive data on the TVPS-R and Figure 1 for a visual depiction of the results.

**Table 2:** **TVPS-R performance by group**.

**Characteristics**	**Controls**	**RD**	**Comorbid**	**ADHD**			
	***M (SD)***	***M (SD)***	***M (SD)***	***M (SD)***	***F*_(3, 259)_**	**Partial η2**	***p*-values**
Discrimination	103.47 (17.52)	99.31 (16.74)	94.32 (16.74)	97.67(16.41)	3.13	0.04	0.026
[95% Confidence intervals]	[99.57–107.38]	[94.70–103.93)	[89.72–98.91]	[94.23–101.11]			
Visual memory	101.76 (17.50)	94.69 (16.82)	90.68 (16.65)	97.64 (16.39)	4.39	0.05	0.005
	[97.86–105.66]	[90.08–99.30]	[86.08–95.27]	[94.20–101.08]			
Visual sequential memory	105.26 (18.72)	95.70 (17.88)	86.89 (17.81)	98.74 (17.52)	10.17	0.11	< 0.001
	[101.09–109.44]	[90.77–100.63]	[81.97–91.80]	[95.06–102.42]			

Means presented in this table are marginal means controlling for WISC-III/-IV VCI.

Two hierarchical multiple regressions were used to determine how well visual processing (entered in step 3) predicted basic reading performance when controlling for VCI (step 1) and either phonological or orthographic processing (step 2). The first regression revealed that, after controlling for VCI and phonological awareness (Elision), visual processing predicted basic reading performance, adjusted *R*^2^ = 0.55, *F*_(5, 256)_ = 64.67, *p* < 0.001. Visual Sequential Memory positively predicted basic reading performance, but Visual Discrimination and Visual Memory were not significant. As expected, VCI and Elision also were significant see Table 3.

**Table 3:** **Hierarchical regression predicting basic reading performance when controlling phonological awareness**.

	**Δ*R*^2^**	***df***	**Δ*F***	***p*-values**
Model 1	0.28	(1, 260)	100.7	< 0.001
	**β**	***t*****-values**	***p*****-values**	
VCI	0.53	10.04	< 0.001	
	**Δ*****R***^2^	***df***	**Δ*****F***	***p*****-values**
Model 2	0.26	(1, 259)	147.37	< 0.001
	**β**	***t*****-values**	***p*****-values**	
VCI	0.30	6.52	< 0.001	
Elision	0.56	12.14	< 0.001	
	**Δ*****R***^2^	***df***	**Δ*****F***	***p*****-values**
Model 3	0.02	(3, 256)	3.39	0.019
	**β**	***t*****-values**	***p*****-values**	
VCI	0.28	6.06	< 0.001	
Elision	0.52	10.75	< 0.001	
TVPS discrimination	–0.01	–0.19	0.85	
Visual memory	–0.03	–0.58	0.57	
Visual sequential memory	0.16	3.12	0.002	

The second regression revealed that, after controlling for VCI and the orthographic processing composite, visual processing predicted basic reading performance, adjusted *R*^2^ = 0.55, *F*_(5, 255)_ = 63.43, *p* < 0.001. Specifically, Visual Sequential Memory positively predicted basic reading performance, whereas Visual Discrimination and Visual Memory were not significant. As expected, VCI and orthographic processing were significant as well see Table 4.

**Table 4:** **Hierarchical regression predicting basic reading performance when controlling orthographic functioning**.

	**Δ*R*^2^**	***df***	**Δ*F***	***p*-values**
Model 1	0.28	(1, 259)	99.11	< 0.001
	**β**	***t*****-values**	***p*****-values**	
VCI	0.53	9.96	< 0.001	
	**Δ*****R***^2^	***df***	**Δ*****F***	***p*****-values**
Model 2	0.25	(1, 258)	133.28	< 0.001
	**β**	***t*****-values**	***p*****-values**	
VCI	0.32	6.96	< 0.001	
Orthographics	0.54	11.55	< 0.001	
	**Δ*****R***^2^	***df***	**Δ*****F***	***p*****-values**
Model 3	0.03	(3, 255)	5.95	0.001
	**β**	***t*****-values**	***p*****-values**	
VCI	0.28	6.02	< 0.001	
Orthographics	0.49	10.69	< 0.001	
TVPS discrimination	0.01	0.2	0.84	
Visual memory	0.01	0.1	0.91	
Visual sequential memory	0.18	3.65	< 0.001	

Prior research has suggested that visual processing and phonological processing may independently contribute to reading ability (Germano et al., 2014), and that memory for order and memory for item may independently contribute to reading ability (Martinez Perez et al., 2012), which is consistent with the notion that the etiology of DD is heterogeneous (Pennington, 2006). As Visual Sequential Memory measures memory for order, one might expect children without phonological awareness deficits to score worse on Visual Sequential Memory than those with phonological awareness deficits, at least in RD, provided the RD groups are low in children who have both deficits or neither. To test this, independent sample *t*-tests were run comparing those with and without phonological awareness deficits on Visual Sequential Memory. A deficit in phonological awareness was determined when the individual scored more than 1.5 *SD* below the mean on Elision. Independent sample *t*-tests were performed twice: once for those with reading disorders (RD and RD/ADHD) and once for those without RD (ADHD and controls). In those with RD, the *t*-test was significant [*t*_(38.27)_ = −2.26, *p* = 0.03] such that children with phonological awareness deficits (*n* = 17, *M* = 100.12, *SD* = 11.45) performed better than those without phonological awareness deficits (*n* = 73, *M* = 92.16, *SD* = 18.51) on Visual Sequential Memory. In children without RD the *t*-test also was significant [*t*_(16.36)_ = 2.11, *p* = 0.05]; however, those with phonological awareness deficits (*n* = 10, *M* = 98.20, *SD* = 7.19) also performed worse on Visual Sequential Memory than those without phonological awareness deficits (*n* = 149, *M* = 103.79, *SD* = 16.50). The interaction approached significance [*F*_(1, 245)_ = 3.71, *p* = 0.055] despite the small *n* of those with phonological awareness deficits using this definition.

## Discussion

Taken together, our findings suggest that children with RD or ADHD have equivalent visual processing to controls on areas assessed that do not require STM for order: visual discrimination and visual STM for complex geographic figures presented singularly. Nonetheless, visual processing may be affected more globally in the comorbid group as they performed worse than controls on all three areas assessed. In terms of STM for order, RD was specifically affected in that children with RD (RD and comorbid RD/ADHD) performed worse than controls, and children with comorbid RD/ADHD performed worse than children with ADHD.

Our findings did not fully support our first hypothesis in that both visual discrimination and visual STM for complex geometric figures presented singularly were comparable to controls for the RD group. We expected that visual STM for the complex figures would be intact but not visual discrimination based upon the literature reviewed (see Kavale and Forness, 2000; Kibby, 2012). The null finding for visual discrimination could be related to power, but our RD mean was very near the expected 100 based on test norms, so even with the addition of more subjects our findings are unlikely to change substantially. It also could be due to our use of verbal IQ as a covariate, as Kavale and Forness found the contribution of perception to reading ability was negligible when they used IQ as a covariate. Nonetheless, our IQ measure assessed verbal intellect orally and did not use pictures. Kavale and Forness' significant findings on discrimination when not controlling IQ could be because the studies upon which the meta-analysis were based included children with comorbid RD/ADHD. The deficits from the ADHD could have led to worse performance on many of the visual measures their studies assessed. In addition, it could be that some of the visual discrimination tasks in the Kavale and Forness study were timed, as processing speed is commonly affected in RD (Shanahan et al., 2006; Peng et al., 2013). Our measure of discrimination was untimed. Based upon our findings, it appears that untimed visual discrimination and visual STM for complex geometric figures presented singularly are intact when using a community sample and ADHD is not comorbid. Although the mean of the RD group on Visual Memory was not 100, our finding STM for complex figures in RD being comparable to typically developing controls is consistent with many other studies focused on visual STM for item in RD/dyslexia (Kibby et al., 2004; Smith-Spark and Fisk, 2007; Kibby and Cohen, 2008; Bacon and Handley, 2014; see Kibby, 2012).

In contrast to the RD group, children with comorbid RD and ADHD (RD/ADHD) had worse performance than controls on both visual discrimination and visual memory, along with worse performance on STM for order, displaying more global problems. As it is believed that ours is the first study to contrast comorbid RD/ADHD to both RD and ADHD alone on these measures; this is a novel finding and worthy of further study. There are at least two potential sources of this more global deficit: poor visual processing in the comorbid group and/or worse focused attention/concentration in the comorbid group that affected their performance on the visual measures. Prior research on RD/ADHD has suggested that having comorbid RD and ADHD is additive, such that having both disorders could lead to worse deficits in the areas affected by either disorder (e.g., phonological processing, working memory, attention), but it is less likely to cause additional deficits unique to RD/ADHD (Willcutt et al., 2005). This would suggest that the weaknesses found in visual discrimination and visual STM for item in the RD/ADHD group are due to focused attention/concentration problems, a common deficit in ADHD, as opposed to a visual deficit not commonly present in either disorder. As noted in the literature review, non-spatial visual processing (perception and STM) is often intact in ADHD (Aman et al., 1998; Kibby and Cohen, 2008; Johnson et al., 2010; Kibby, 2012), and we found it to be spared in our ADHD sample. Furthermore, our RD sample also performed comparably to controls on discrimination and visual STM for complex figures. Thus, we believe the weaknesses in visual discrimination and visual STM for item in the comorbid group are due to worse inattention as opposed to visual processing deficits unique to this group. To test this we examined Pearson correlations between TVPS Discrimination and Visual Memory and WISC-III/WISC-IV Picture Completion (a measure of visual processing that minimizes spatial processing) and BASC/BASC-2 Attention Problems. TVPS-R Discrimination was not correlated with Picture Completion (*r* = 0.08, *p* = 0.22), but it was significantly correlated with Attention Problems (*r* = −0.21, *p* = 0.001). TVPS-R Visual Memory was correlated with Picture Completion (*r* = 0.14, *p* = 0.03), but it had a larger correlation with Attention Problems (*r* = −0.24, *p* < 0.001). Furthermore, all four groups were comparable on Picture Completion [*F*_(3, 242)_ < 1.0, *p* = 0.48], but they differed on Attention Problems [*F*_(3, 242)_ = 41.86, *p* < 0.001], with children with comorbid ADHD/RD performing worse than controls and RD. Thus, it is likely that attention problems were related to the visual processing weaknesses found in the comorbid group. Future research should examine alternate measures of visual processing and attention problems to determine if these relations will hold.

Consistent with the first hypothesis, both reading disorder groups performed worse than controls on the visual STM task requiring memory for order (TVPS-R Visual Sequential Memory), but the ADHD group did not. Moreover, the comorbid group also performed significantly worse than the ADHD group, demonstrating that the weakness was specific to RD. Nonetheless, there may be slight additive effects in that the RD/ADHD tended to perform worse than the RD group, although this was not significant (*p* = 0.07). Given the trend found, it is worthy of further study. Visual Sequential Memory measures memory for order, as the participant must select the item choice that presents the shapes in the correct order. In addition, this task may tap linguistic skills as some children may enhance their performance by using verbal labels to help remember the shape order. Thus, a deficit in memory for order and/or linguistic processing in the RD groups may be driving this finding. Majerus et al. have demonstrated that deficits in memory for order are separable from deficits in memory for item in RD using verbal tasks and that deficits in memory for order are related to reading ability (Martinez Perez et al., 2012; Perez et al., 2012). Various linguistic deficits are common in RD as well (see Pennington, 2008 for a review). Using Pearson correlations we found vocabulary knowledge WISC-III/IV Vocabulary) was correlated with Sequential Memory (*r* = 0.32, *p* < 0.001) as was a verbal measure of memory for order, Sentence Memory, which requires verbatim repetition (*r* = 0.23, *p* < 0.001). Thus, both linguistic deficits (sematic/lexical) and memory for order/sequential processing deficits may be playing a role in the deficit found on Sequential Memory. The specificity of the sequential STM weakness to RD helps to explain why some children have RD but not ADHD despite the relatively high comorbidity between the two disorders. There are other deficits that are specific to RD as well, such as poor phonological processing (Pennington, 2008; Willcutt et al., 2010), whereas slow processing speed may be a source of their comorbidity (Willcutt et al., 2005; Shanahan et al., 2006).

When assessing the total sample, only sequential STM predicted basic reading ability of the various visual processes, explaining ~2–3% of the variance after controlling verbal intellect and phonological processing, which is consistent with the second hypothesis, or verbal intellect and orthographic processing, which is not consistent with the second hypothesis. It was expected that sequential STM would predict basic reading ability, even when controlling for phonological processing ability, as visual processing and phonological processing deficits can be independent of each other in RD (Bosse et al., 2007; Lassus-Sangosse et al., 2008; Germano et al., 2014). Furthermore, as noted in the literature review, visual processing likely contributes to the orthographic route more strongly than the phonological route (Boder, 1973; Corcos and Willows, 1993; Stein and Talcott, 1999; Au and Lovegrove, 2006; Mesman and Kibby, 2011). Thus, we did not expect visual processing to predict basic reading ability when orthographic processing was controlled. Nonetheless, reduced verbal (semantic/lexical) mediation of the task in the RD groups may have driven this relationship, which may explain why Visual Sequential Memory predicted basic reading ability regardless of whether phonological or orthographic processing was controlled. Another possible explanation is that reduced memory for order may have driven this relationship (Perez et al., 2012; Martinez Perez et al., 2013). Thus, further testing of these relationships is required to determine which contributor(s) to Visual Sequential Memory is driving these findings.

Contrary to the second hypothesis, visual discrimination did not predict basic reading ability when phonological processing was controlled. It also did not predict basic reading ability when orthographic processing was controlled. Our null findings on discrimination could be due to two factors. The first is that untimed discrimination may not be affected, and thus not contribute much to reading variability, when most individuals in the sample have deficits specific to RD and/or ADHD or have neither disorder. The other is that we controlled verbal intellect (VCI), and Kavale and Forness (2000) noted that the perceptual factors they assessed, including visual discrimination, contributed minimally to reading ability when IQ was controlled. Nonetheless, the IQ measures from the studies they used may have required visual discrimination for some of the tests/subtests, which is why we used verbal intelligence as the covariate. To determine if controlling VCI was the reason why discrimination was not significant, we re-ran the regression equations without using it in the equations. Visual Discrimination did not predict reading ability regardless of whether phonological awareness was used in step 1, orthographic processing was used in step 1, or neither (just the three TVPS-R measures were entered), *p*s > 0.10. Furthermore, it was not a good predictor (*p* = 0.83) of reading ability when the sample was comprised solely of children with RD/ADHD and only the three TVPS-R measures were entered. Hence, untimed discrimination does not appear to be a good predictor of reading ability when one is using a community sample.

We conducted an exploratory analysis to determine whether children with RD but not phonological awareness problems performed worse than children with RD and phonological awareness problems on sequential STM. It was believed that this may be the case based upon articles which suggested that dyslexia could arise from either phonological awareness problems or visual processing problems (e.g., Bosse et al., 2007; Lassus-Sangosse et al., 2008; Germano et al., 2014) or poor memory for order (Perez et al., 2012; Martinez Perez et al., 2013). Hence, individuals with dyslexia and phonological awareness problems may have good sequential STM, and those with dyslexia but intact phonological awareness may have weaker sequential STM. This is what we found. For children with RD, those with impaired phonological processing performed better on Visual Sequential Memory than those with spared phonological processing. Hence, our findings support the work of prior researchers suggesting the deficits in phonological processing and sequential memory are dissociable in RD (Perez et al., 2012; Martinez Perez et al., 2013). Of interest, children without RD had the opposite results; those with poor phonological processing performed worse on Visual Sequential Memory than those without poor phonological processing. Thus, there may be something specific about the relationship between phonological processing and sequential STM to RD that is not found in controls and ADHD. Further research in this area is warranted.

Although our study yielded several interesting findings, it has various limitations that future research should address. One limitation is that we had a mild sample, in terms of both RD and ADHD severity, as it was a community sample. We may have found deficits on more measures or more severe deficits if we had a clinic sample. At least it appears that in a community sample discrimination of complex figures is intact in RD and ADHD, and the sequential STM deficit may be specific to RD. Another limitation is that the number of visual processing measures we used was limited. We used the same instrument to assess the various visual processing constructs (TVPS-R) for this study, which facilitates direct comparisons amongst the measures as their scores were based on the same normative sample. In addition, we were only able to administer three subtests of the TVPS-R for time reasons. Our battery for the larger studies from which this one was drawn was quite extensive, requiring us to focus on what we believed to be the most crucial measures. As visual discrimination and visual STM were processes that Boder (1973) originally speculated to be crucial to dyslexia of the dyseidetic (visual) type, and as visual discrimination and visual STM were areas Kavale and Forness (2000) later found to be the most consistently related to various aspects of reading amongst those with and without RD, we chose these measures to be part of our test battery. Future research should include a measure of visual closure as, depending upon the analysis, Kavale and Forness also found it to be modestly predictive of reading performance and Germano et al. (2014) found it to be affected in their DD group. In addition, future research should include other measures of visual processing such as visual attention (Valdois et al., 2004; Bosse et al., 2007) and visual sequential STM tasks that do not use labelable stimuli.

In summary, visual processing may be intact in RD and ADHD when measured with tasks of untimed discrimination and visual STM that do not require sequential processing or allow easy labeling. Although more general visual processing deficits were found in the comorbid group, it is likely these were due to worse focused attention/concentration caused by the additive effects of having both RD and ADHD rather than additional visual processing problems unique to this group *per se.* Deficits in sequential STM were specific to reading disorders, with weaknesses being found in both the RD group and the comorbid RD/ADHD group. Moreover, sequential STM was modestly related to basic reading performance even after controlling for orthographic and phonological processing skills, but discrimination and visual STM were not. Further research is needed to determine whether the findings related to Visual Sequential Memory were due to sequential processing deficits in the RD group, such as poor memory for order, and/or reduced verbal mediation of the task by the RD group due to linguistic weaknesses. Based upon our exploratory analyses, we believe it to be due, at least in part, to both weaknesses. Our study is among the first to show that reduced memory for order using a visual task is specific to RD, with the comorbid group being particularly affected. Thus, replication of this finding is warranted, using both verbal and visual STM tasks, to determine if there are effects specific to modality.

### Conflict of interest statement

The authors declare that the research was conducted in the absence of any commercial or financial relationships that could be construed as a potential conflict of interest.
